# HBx-related long non-coding RNA DBH-AS1 promotes cell proliferation and survival by activating MAPK signaling in hepatocellular carcinoma

**DOI:** 10.18632/oncotarget.5667

**Published:** 2015-09-15

**Authors:** Jin-lan Huang, Ting-yu Ren, Shun-wang Cao, Shi-hao Zheng, Xiu-mei Hu, Yan-wei Hu, Li Lin, Jing Chen, Lei Zheng, Qian Wang

**Affiliations:** ^1^ Laboratory Medicine Center, Nanfang Hospital, Southern Medical University, Guangzhou, Guangdong, China; ^2^ Department of Neurosurgery, Fujian Provincial Hospital, Fuzhou, Fujian, China

**Keywords:** lncRNA, DBH-AS1, HCC, proliferation, HBx

## Abstract

Accumulating evidence supports an important role for the hepatitis B virus x protein (HBx) in the pathogenesis of hepatitis B virus (HBV)-induced hepatocellular carcinoma (HCC), but the underlying mechanisms are not entirely clear. Here, we identified a novel long noncoding RNA (lncRNA) DBH-AS1 involved in the HBx-mediated hepatocarcinogenesis. The levels of DBH-AS1 were positively correlated with hepatitis B surface antigen (HBsAg) and tumor size in HCC tissues. Functionally, transgenic expression of DBH-AS1 significantly enhanced cell proliferation and tumorigenesis, whereas short hairpin RNA knockdown of DBH-AS1 caused an inhibition of cell proliferation. Mechanistically, overexpression of DBH-AS1 induced cell cycle progression by accelerating G1/S and G2/M transition concomitantly with upregulation of CDK6, CCND1, CCNE1 and downregulation of p16, p21 and p27. We also found that enhanced DBH-AS1 expression inhibited serum starvation-induced apoptosis of HCC cells. In contrast, suppressed DBH-AS1 expression had opposite effects. Furthermore, DBH-AS1 was shown to activate MAPK pathway. We also provide evidence that DBH-AS1 could be significantly induced by HBx protein and markedly down-regulated by p53. Thus, we concluded that DBH-AS1 can be induced by HBx and inactivated by p53, and consequently promote cell proliferation and cell survival through activation of MAPK signaling in HCC. Our study suggests that DBH-AS1 acts as an oncogene for HCC.

## INTRODUCTION

Hepatocellular carcinoma (HCC), accounting for 90% of primary liver cancers, is a highly lethal cancer with increasing worldwide incidence [1]. There are more than 250 000 new HCC cases and estimated 500 000-600 000 deaths due to this disease annually [1-3]. Despite of the therapeutic advances that had been made in HCC in the past few decades, such as surgical resection, liver transplantation, and adjuvant therapy, the overall 5-year survival rate of HCC patients still remains poor [4]. The major risk factor for HCC is chronic infection with hepatitis B virus (HBV), which accounts for 52% of all HCC [5]. HBV X protein (HBx), encoded by HBV x gene, has been implicated to act as a multifunctional oncogenic factor in the development of HBV-related HCC, including promoting cell cycle progression, inactivating negative growth regulators, regulating apoptosis and inhibiting nucleotide excision repair of damaged cellular DNA [6]. However, the molecular mechanisms underlying HBx protein-mediated tumorigenesis are not entirely clear.

Long non-coding RNAs (lncRNAs) are defined as transcribed RNA molecules greater than 200nt in length with limited or no protein-coding capacity [7]. Recent studies have demonstrated that various lncRNAs, such as HULC, ATB, HOTAIR, HOTTIP, URHC, Dreh, UFC1, can participate in diverse biological processes involved in hepatocarcinogenesis, including cell proliferation, apoptosis and metastasis [8-11]. Importantly, several lncRNAs have been identified to be related to HBx protein. Huang, J. F. et al [12] characterized a lncRNA Dreh in mouse which could be down-regulated by HBx protein. Dreh was elucidated to inhibit HCC metastasis by targeting the intermediate filament protein vimentin. Furthermore, HULC, a highly expressed lncRNA in HCC, was found to be elevated by HBx protein and promote hepatocyte proliferation via down-regulating p18 [13]. Although several lncRNAs have been demonstrated to be regulated by HBx protein, the specific role of HBx-related lncRNAs in HCC remains largely unknown.

Recently, a microarray analysis carried out by Magkoufopoulou, C. et al [14] revealed that quercetin results in decreased expression of lncRNA DBH-AS1 (NCBI Accession NO. NR_102735; UCSC ID uc031tfk.1) in HepG2 cells. DBH-AS1 is a ~2kb lncRNA with a polyadenylated tail transcribed from chromosome 9q34 [15]. Quercetin, the prototype of a naturally-occurring chemopreventive agent for various cancers, has been reported to possess anti-proliferative and antioxidant activities in hepatocytes [16-18]. Hsieh, A. et al [19] found that quercetin effectively represses HBx-mediated regulation of several key oncogenes. However, whether DBH-AS1 is involved in the development of HBV-related HCC has not been elucidated so far.

In this study, we first assessed the levels of DBH-AS1 in 45 HCC tumor tissues. The clinical data of the patients in our study showed that high levels of DBH-AS1 are positively associated with tumor size and hepatitis B surface antigen (HBsAg). Further investigation of the function of DBH-AS1 in HCC revealed that DBH-AS1 promotes cell proliferation and survival. Moreover, activation of the ERK/p38/JNK MAPK signaling pathway is observed in cells overexpressing DBH-AS1. Furthermore, DBH-AS1 is found to be significantly induced by HBx protein and inactivated by p53. Totally, these results suggested that DBH-AS1 exerts an impact as a potential oncogene and may provide us new insight into the role of HBx-related lncRNAs in the development of HCC.

## RESULTS

### Associations between lncRNA DBH-AS1 expression and clinicopathological characteristics

To investigate the role of DBH-AS1 in HCC, we examined DBH-AS1 expression in 45 HCC tumor tissues by qRT-PCR. Statistical analysis revealed that high DBH-AS1 levels were positively correlated with tumor size (χ2 = 8.006, *P* = 0.005) and HBsAg (χ2 = 4.132, *P =* 0.042, Table 1). However, we did not find any correlation between DBH-AS1 levels and other clinicopathological features, including gender, age, AFP level, liver cirrhosis, tumor number and Edmondson grade. These data indicate that DBH-AS1 may be involved in HCC tumor growth and potentially be related to HBV infection.

**Table 1:** Correlation between lncRNA DBH-AS1 expression and clinicopathological characteristics in HCC patients (n=45)

Variables	lncRNA DBH-AS1 expression		x2	*P Value
	Low	High^#^		
**All cases**	22	23		
**Age**			0.218	0.641
**>55**	9	11		
**<=55**	13	12		
**Gender**			0.505	0.477
**Male**	19	18		
**Female**	3	5		
**HBsAg**			4.132	0.042*
**positive**	12	19		
**negative**	10	4		
**Liver cirrhosis**			1.779	0.182
**with**	10	15		
**without**	12	8		
**AFP(ng/ml)**			0.192	0.661
**>20**	11	10		
**<=20**	11	13		
**Tumor size(cm)**			8.006	0.005**
**>5cm**	7	17		
**<=5cm**	15	6		
**Tumor number**			0.505	0.477
**solitary**	19	18		
**multiple**	3	5		
**Edmondson grade**			0.67	0.413
**I+II**	2	4		
**III**	20	19		

# The median expression level was used as the cutoff. Low expression of lncRNA DBH-AS1 in 22 patients was classified as values below the 50th percentile. High lncRNA DBH-AS1 expression in 23 patients was classified as values at or above the 50th percentile.

* For analysis of correlation between lncRNA DBH-AS1 levels and clinical features,Pearson's chi-square tests were used. *, *P* <0.05; **, *P*<0.01.

### LncRNA DBH-AS1 promotes HCC cell proliferation *in vitro*

To evaluate the biological functions of DBH-AS1, the levels of DBH-AS1 in HCC cell lines and hepatic immortal cell lines were analyzed by qRT-PCR. Higher levels of DBH-AS1 were observed in the SK-Hep1, Hep3B, MHCC97H and HepG2 cell lines than in the hepatic immortal cell line LO2 and QSG7701 (Supplementary Figure S1A).

**Figure 1:**
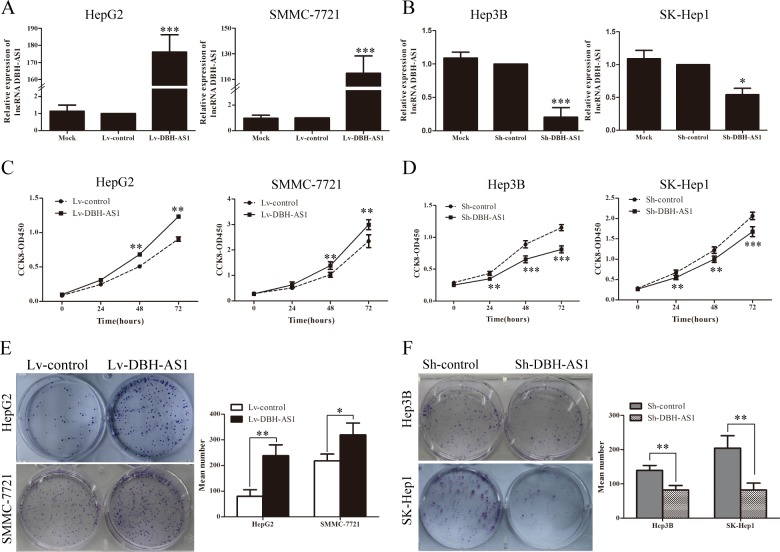
LncRNA DBH-AS1 promotes HCC cell proliferation *in vitro* **A.** HepG2 and SMMC-7721 cells were infected with lentivirus carrying the DBH-AS1 gene, and HepG2 and SMMC-7721 cells stably overexpressing DBH-AS1 were screened by qRT-PCR. **B.** Short hairpin RNA against DBH-AS1 stably decreased the expression of DBH-AS1 in sh-DBH-AS1 Hep3B and SK-Hep1 cells compared with sh-control cells by qRT-PCR. **C.** After overexpression of DBH-AS1 in HepG2 and SMMC-7721 cells, the cell viability was assessed by CCK-8 assays daily for 3 days. **D.** Cell viability was assessed by CCK-8 assays daily for 3 days in Hep3B and SK-Hep1 cells with silenced DBH-AS1 expression. **E.** Colony formation assays were performed on HepG2 and SMMC-7721 cells stably overexpressing DBH-AS1 for 2 weeks. **F.** *In vitro* proliferative ability of Hep3B and SK-Hep1 cells was significantly decreased in DBH-AS1-suppressed cells compared to sh-control cells by colony formation assays. Data are presented as mean ± SD for at least three independent experiments, *P < 0.05, **P < 0.01, ***P < 0.001.

To further investigate the biological effect of DBH-AS1 in HCC cells, we constructed HepG2 and SMMC-7721 cell lines with stably overexpressed DBH-AS1, and Hep3B and SK-Hep1 cell lines with stably silenced DBH-AS1 expression (Figure 1A). The growth curves determined by CCK-8 assays indicated that cell proliferation was enhanced by overexpression of DBH-AS1 in HepG2 and SMMC-7721 cells, whereas knockdown of endogenous DBH-AS1 expression dramatically reduced the proliferative capacity of Hep3B and SK-Hep1 cells (Figure 1B). Further colony formation assays also showed that up-regulation of DBH-AS1 could significantly enhance the colony formation ability in HepG2 and SMMC-7721 cells. In contrast, suppressed DBH-AS1 expression had the opposite effect (Figure 1C). Thus, these data revealed that DBH-AS1 enhances HCC cell growth.

### LncRNA DBH-AS1 promotes tumor growth *in vivo*

**Figure 2:**
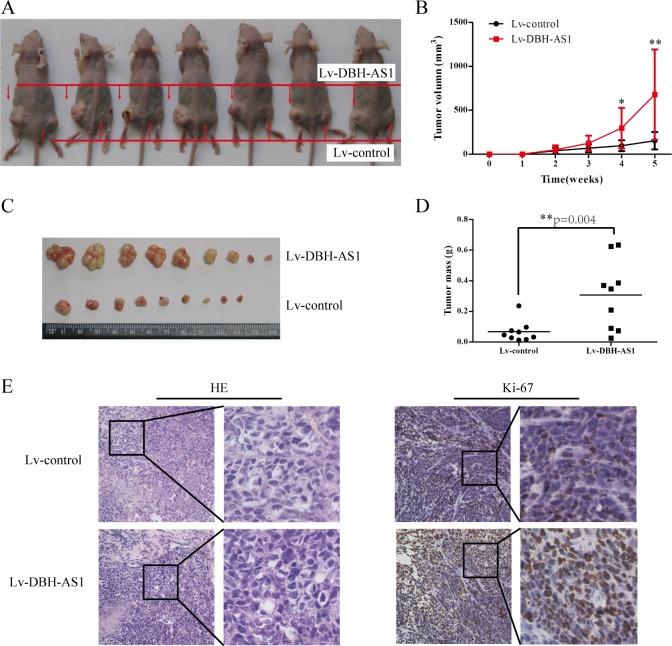
LncRNA DBH-AS1 accelerates tumor growth *in vivo* **A.** Photographs of tumors that developed in xenograft-transplanted nude mouse tumor models 5 weeks after injection of DBH-AS1-overexpressing or control SMMC-7721 cells. **B.** *In vivo* subcutaneous tumor growth curves were shown for SMMC-7721 cells of Lv-DBH-AS1 and Lv-control vectors. Images **C.** and weights **D.** of xenografts established by subcutaneous transplantation with Lv-DBH-AS1-overexpressing and Lv-control SMMC-7721 cells 5 weeks after cell injection. **E.** H&E-stained paraffin-embedded sections obtained from xenografts. IHC staining shows that the expression of Ki67 was enhanced in the Lv-DBH-AS1 group compared to the Lv-control group. The higher magnification for the selected region in each part was shown in the right of each part. Original magnification 400×.

To determine the growth-enhancing effect of DBH-AS1 *in vivo*, we injected SMMC-7721 cells stably transfected with Lv-DBH-AS1 or Lv-control subcutaneously into nude mice for xenotransplantation. Mice injected with cells overexpressing DBH-AS1 showed significantly increased tumor growth compared to those injected with cells transfected with Lv-control (Figure 2A). As assessed by measurements of tumor volume and mass, enhanced DBH-AS1 expression significantly promoted overall tumor growth (Figure 2B-2D). The immunohistochemistry analysis of the tumor tissues from xenografts revealed that the expression of Ki67 proliferation antigen was significantly stronger in xenografts of Lv-DBH-AS1 cells than in xenografts of Lv-control cells (Figure 2E). These results showed that DBH-AS1 accelerates HCC cell proliferation *in vivo*.

### LncRNA DBH-AS1 induces cell-cycle progression in HCC cells

**Figure 3:**
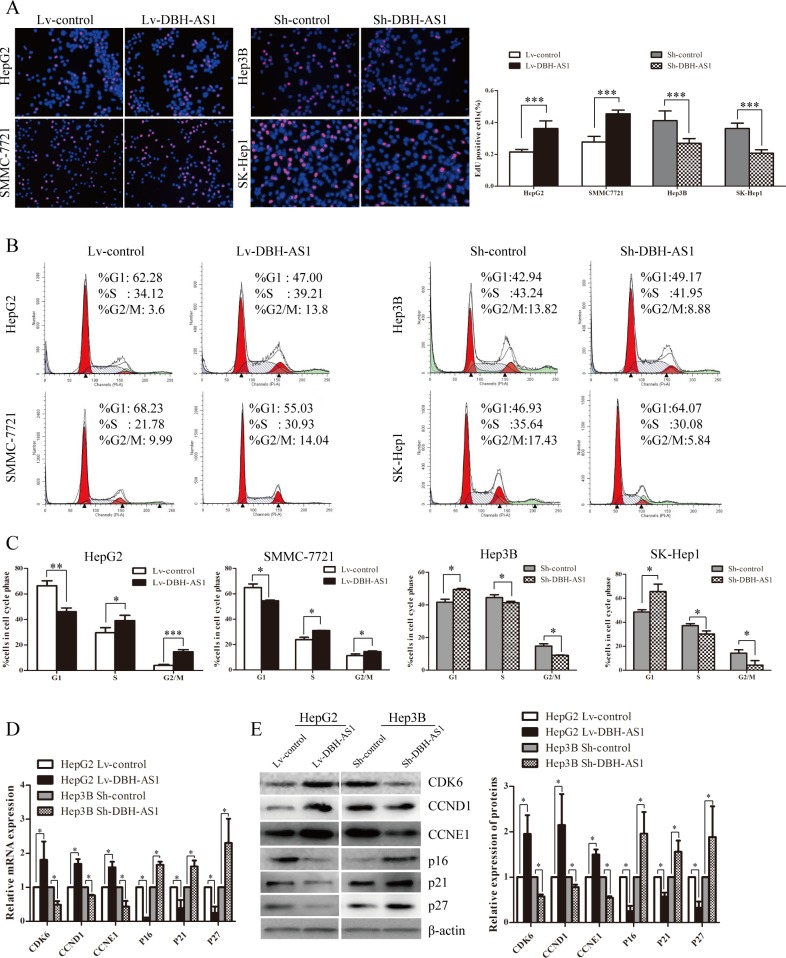
LncRNA DBH-AS1 induces cell-cycle progression in HCC cells **A.** HepG2 and SMMC-7721 cells with elevated DBH-AS1 expression were seeded on 96-well plates, and cell proliferation was examined by EdU immunofluorescence staining. Effect of DBH-AS1 knockdown on Hep3B and SK-Hep1 cell proliferation was also measured by EdU immunofluorescence staining. The graph on the right shows the percentage of EdU-positive nuclei. **B.** Cell-cycle analysis of HepG2 and SMMC-7721 cells overexpressing DBH-AS1 and Hep3B and SK-Hep1 cells with stably silenced DBH-AS1 expression. **C.** Proportion of cells in various phases of the cell cycle. **D.**-**E.** The relative expression levels of cell cycle associated genes, including CDK6, CCND1, CCNE1, P16, P21 and P27, were detected in HepG2 cells overexpressing DBH-AS1 and Hep3B cells with stably down-regulated DBH-AS1 expression by qRT-PCR **D.** and western blot with quantitative analysis **E.**. The results show the means ± SD from at least 3 separate experiments. *P < 0.05, **P < 0.01, ***P < 0.001.

To gain insights into the mechanism by which DBH-AS1 enhances HCC cell proliferation, EdU incorporation assays and fluorescence-activated cell sorting (FACS) were performed to analyze differences in cell-cycle distributions after DBH-AS1 overexpression or silencing. EdU incorporation assays showed that overexpression of DBH-AS1 significantly increased the percentage of EdU positive cells in HepG2 and SMMC-7721 cells whereas DBH-AS1 depletion resulted in a marked reduction in the percentage of EdU positive cells in Hep3B and SK-Hep1 cells, indicating that DBH-AS1 facilitates the entry of cells into S phase (Figure 3A). Consistent with our EdU results, a reduction in the G1 population and an increase in the S and G2/M population were observed in HepG2 and SMMC-7721 cells overexpressing DBH-AS1. Conversely, repressed DBH-AS1 expression in Hep3B and SK-Hep1 cells mainly led to a G1 accumulation and a decrease of S and G2/M phase (Figure 3B and 3C). Furthermore, we also examined the levels of several key genes involved in cell cycle checkpoint in HepG2 cells stably overexpressing DBH-AS1 and Hep3B cells with silenced DBH-AS1 expression by qRT-PCR and western blot analysis. Overexpression of DBH-AS1 in HepG2 cells elevated the expression of oncogenic cell-cycle regulators including CDK6, CCND1, CCNE1, but reduced the expression of cyclin-dependent protein kinase inhibitors including p16, p21, p27 (Figure 3D and 3E). By contrast, knockdown of DBH-AS1 in Hep3B cells resulted in a decreased expression of CDK6, CCND1, CCNE1 and an increased expression of p16, p21, p27 (Figure 3D and 3E).

### LncRNA DBH-AS1 inhibits serum starvation-induced apoptosis of HCC cells

**Figure 4:**
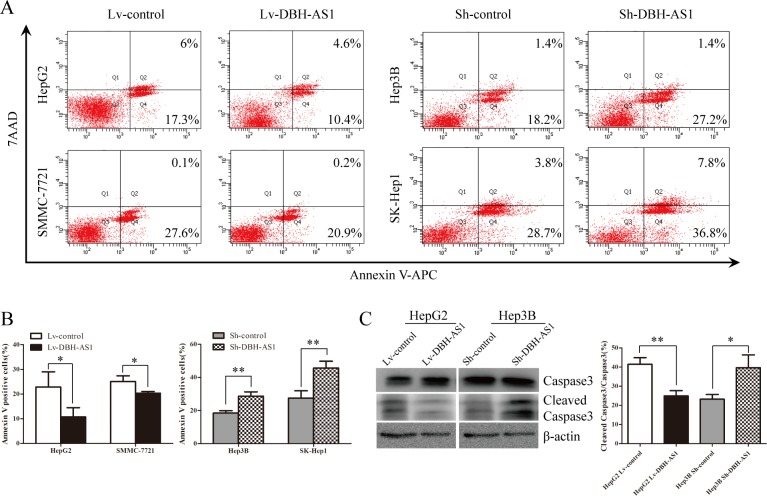
LncRNA DBH-AS1 inhibits serum starvation-induced apoptosis of HCC cells **A.** Cells were cultured in serum-free medium for 48h. The apoptosis rate was measured by FACS-based Annexin-V/7AAD double staining. Cells positive for annexin V staining were counted as apoptotic cells. **B.** The bar graph shows the percentage of apoptotic cells. **C.** The levels of Caspase3 and cleaved Caspase3 were detected by western blot with quantitative analysis in HepG2 cells with elevated DBH-AS1 expression and Hep3B cells with silenced DBH-AS1 expression. The experiments were performed in triplicate; the data are expressed as the mean ± SD. *P < 0.05, **P < 0.01, ***P < 0.001.

Because DBH-AS1 exerts an oncogenic effect in HCC cells, we speculated that DBH-AS1 may be critical for cell survival and apoptosis. To test this hypothesis, we further checked the effect of DBH-AS1 on HCC cell survival. Apoptosis was measured by FACS-based Annexin-V/7-AAD double staining in HCC cells under serum starvation condition for 48h. The results revealed that the percentage of Annexin V-positive cells was lower in DBH-AS1-overexpressing HepG2 and SMMC-7721 cells than control cells. In contrast, sh-DBH-AS1 Hep3B and SK-Hep1 cells had a significantly higher percentage of Annexin V-positive cells than sh-con group (Figure 4A and 4B). We further examined the expression of 2 well-defined apoptosis protein markers, caspase 3 and its active form of cleaved caspase 3. The ratio of cleaved caspase 3/caspase 3 was remarkably decreased in HepG2 cells with elevated DBH-AS1 expression, whereas the opposite effects could be found in Hep3B cells with suppressed DBH-AS1 expression (Figure 4C). Collectively, our data suggest that DBH-AS1 inhibits serum starvation-induced apoptosis and enhances HCC cell survival.

### LncRNA DBH-AS1 activate MAPK signaling pathways in HCC cells

**Figure 5:**
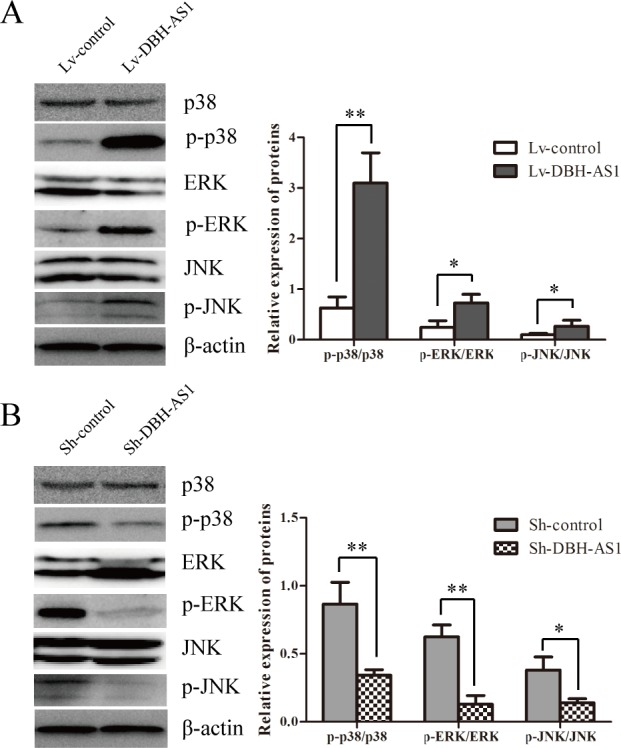
LncRNA DBH-AS1 activates MAPK signaling pathways The levels of ERK, p38, JNK, p-ERK, p-p38 and p-JNK were examined by western blot analysis in HepG2 cells overexpressing DBH-AS1 **A.** and Hep3B cells with silenced DBH-AS1 expression **B.**. The experiments were performed in triplicate; the data are expressed as the mean ± SD. *P < 0.05, **P < 0.01, ***P < 0.001.

Mitogen-activated protein kinases (MAPKs), including extracellular signal-regulated kinase (ERK), c-Jun N-terminal kinase (JNK) and p38, are crucial molecules involved in pathways associated with cancer pathogenesis.[20] Activation of MAPK signaling pathways in HepG2 cells overexpressing DBH-AS1 was first determined by western blot analysis. Higher levels of phospho-ERK (p-ERK), phospho-p38 (p-p38) and phosphor-JNK (p-JNK) MAPK were observed in DBH-AS1 overexpressing HepG2 cells than in control cells (Figure 5A). By contrast, inhibition of DBH-AS1 in Hep3B cells strongly reduced the levels of p-ERK, p-p38 and p-JNK MAPK (Figure 5B).

### LncRNA DBH-AS1 is significantly induced by HBx

**Figure 6:**
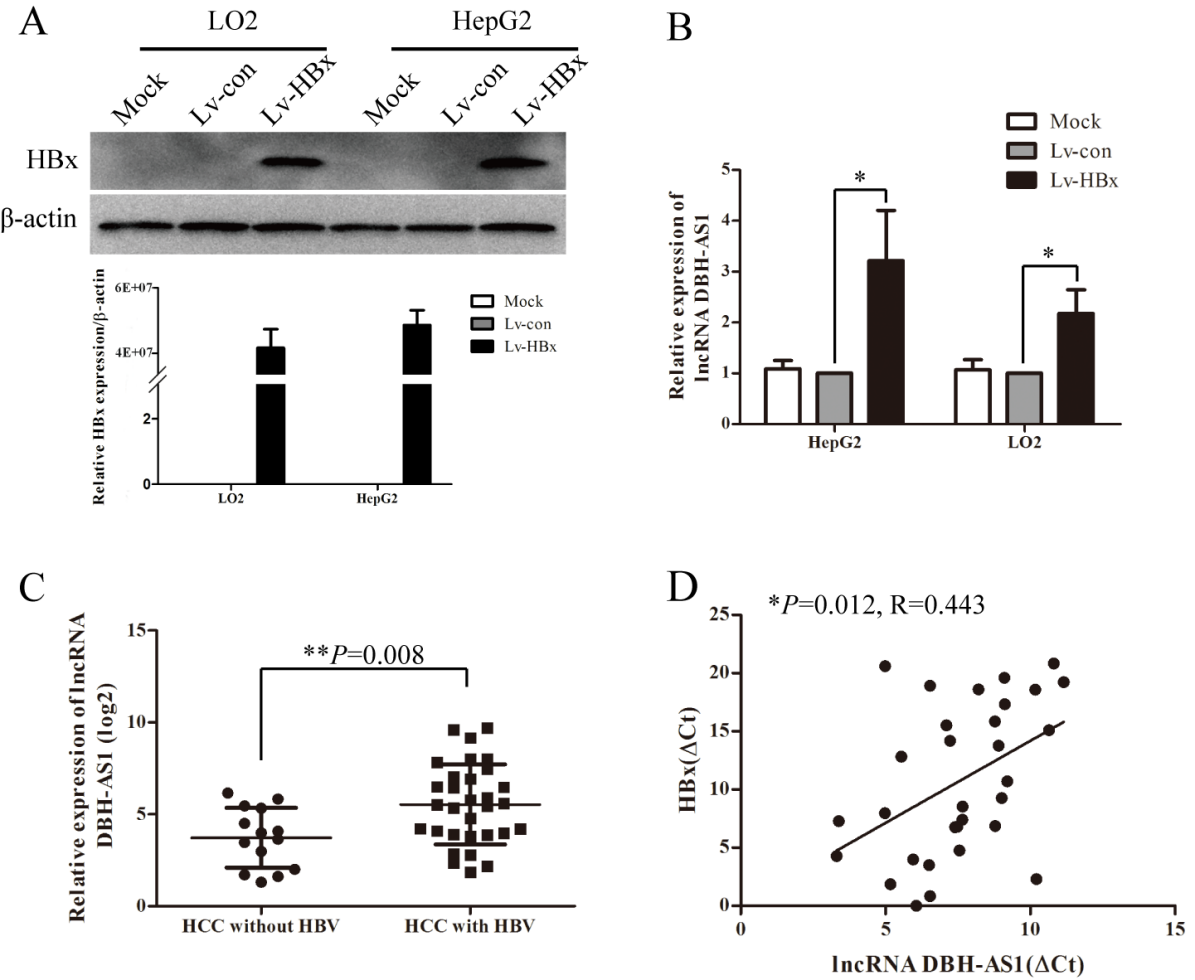
HBx induces the expression of lncRNA DBH-AS1 **A.** Ectopic re-expression of HBx was detected in Lv-HBx-transfected HepG2 and LO2 cells by qRT-PCR and western blot. β-actin was used as a loading control. **B.** The relative expression of lncRNA DBH-AS1 in HepG2 and LO2 cells re-expressing HBx compared with controls by qRT-PCR. Data are shown as the mean±SD based on at least three independent experiments. **C.** Comparison of levels of DBH-AS1 in HCC patients with and without HBV infection (independent *t* test). **D.** The correlation between DBH-AS1 transcript level and HBx mRNA level in 31 HCC tissues. The ΔCt values were subjected to Pearson correlation analysis. *P < 0.05, **P < 0.01, ***P < 0.001.

Since clinical data showed that the expression of DBH-AS1 was positively correlated with HBsAg, it is possible that HBV infection would influence the expression of DBH-AS1. Thus, we constructed cell lines that stably re-expressing HBx by lentivirus infection of hepatic immortal cell line LO2 cells and HCC cell line HepG2 cells (Figure 6A). We noticed that DBH-AS1 was significantly up-regulated in cells stably re-expressing HBx in comparison with the control group (Figure 6B). Importantly, levels of DBH-AS1 were higher in HCC patients with HBV infection (31 cases) than those without HBV infection (14 cases) (*P* = 0.008, Figure 6C). Furthermore, to confirm the regulation of DBH-AS1 by HBx in human HCC tissues, we measured DBH-AS1 transcript levels and HBx mRNA levels in the same set of 31 HCC tissues with HBV infection by qRT-PCR. The results showed that DBH-AS1 transcript level was positively correlated with HBx mRNA level (*R* = 0.443, *P* = 0.012, Figure 6D).

### LncRNA DBH-AS1 is inactivated by p53

**Figure 7:**
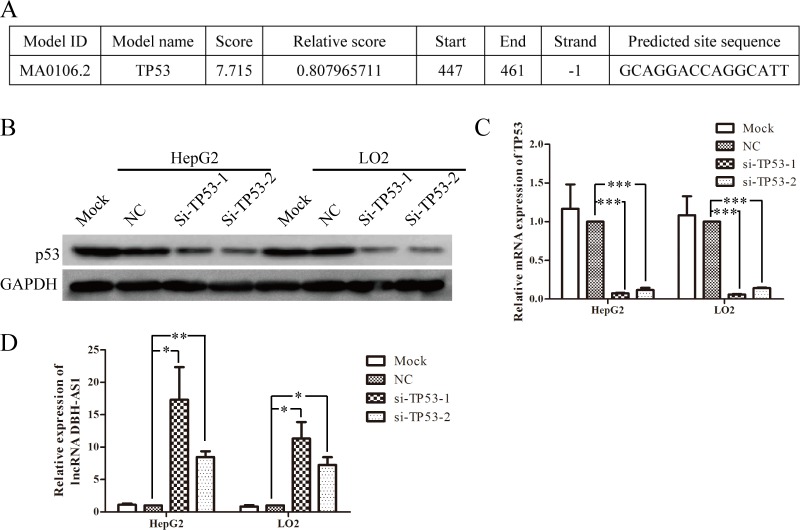
LncRNA DBH-AS1 is inactivated by p53 **A.** The potential p53-binding site upstream of DBH-AS1 predicted by JASPAR database. **B.** Western blot analysis showed the reduced levels of p53 protein in HepG2 cells and LO2 cells transfected with siRNAs. **C.** Reduced p53 mRNA expression by siRNAs in HepG2 cells and LO2 cells was shown by qRT-PCR. **D.** Expression of DBH-AS1 transcripts was quantified by qRT-PCR. Data shown are the mean ± SD of three independent experiments. *P < 0.05, **P < 0.01, ***P < 0.001.

To investigate the regulator upstream of DBH-AS1, the online JASPAR database (http://jaspar.genereg.net/) was used to analyze the 2000-bp sequence upstream of DBH-AS1 gene. A putative p53-binding site spanned the −447 to −461bp positions (Figure 7A). Thus, we addressed whether DBH-AS1 is mediated by P53. The expression of P53 in HepG2 and LO2 cells was down-regulated by transfection with siRNAs targeting the TP53 gene. SiRNAs significantly decreased p53 mRNA and protein levels in HepG2 cells and LO2 cells (Figure 7B and 7C). Meanwhile, DBH-AS1 levels were significantly elevated in cells transfected with anti-TP53 siRNAs compared to those transfected with a scramble control (Figure 7D). Additionally, it is reported that lncRNA H19 can be regulated by p53 under hypoxia.[21] Interestingly, we also observed that DBH-AS1 was up-regulated under hypoxic stress (Figure S1B and S1C).

## DISCUSSION

HCC is one of the most common cancers worldwide with high prevalence and lethality, which is positively associated with HBV infections [22]. HBx protein encoded by HBV x gene has been elucidated to induce HCC by promoting cell cycle progression and inhibiting the expression of various tumour suppressor genes [6]. Recent studies have indicated that some lncRNAs related to HBx may act as oncogenes or tumor suppressors, thus involved in HCC pathogenesis [12, 13]. Although dysregulation of several HBx-related lncRNAs associated with HCC have been identified, the function and clinical significance of the majority of HBx-related lncRNAs in the progression and aggressiveness of HCC remain unknown.

In this study, we identified a novel HBx-related lncRNA DBH-AS1. Clinical data indicated that high levels of DBH-AS1 were positively correlated with HBsAg and tumor size in HCC patients. Here, we firstly investigated the biological functions of DBH-AS1 by gain-of-function and loss-of-function experiments. We provided evidence that DBH-AS1 promoted HCC cell proliferation *in vitro* and *in vivo*. Mechanistically, DBH-AS1 was shown to induce cell cycle progression by accelerating G1/S and G2/M transition. It has been well-defined that tumour-associated cell cycle defects are often mediated by the accumulation of cyclins (CCNs) and cyclin-dependent kinases (CDKs) complexes and reduced activity of cyclin-dependent kinase inhibitors (CDKIs) [23, 24]. Our data suggest that DBH-AS1 can modulate several cell-cycle related factors, including down-regulating p16, p21 and p27 (members of CDKIs) as well as up-regulating CDK6 (a member of CDKs), CCND1, CCNE1 (members of CCNs). In addition to enhanced proliferation, resistance to apoptosis is also a hallmark of cancer cells [25]. In this study we also found that DBH-AS1 can protect HCC cells from serum starvation-induced apoptosis and promote cell survival. This is the first report to demonstrate the functional significance of DBH-AS1 in HCC, and our results indicate that DBH-AS1 may function as an oncogene. Consistent with our results, Magkoufopoulou, C. et al [14] reported that the anti-cancer drug quercetin can reduce the expression of DBH-AS1 in HepG2 cells, partly in support to our hypothesis.

Currently, ERK/p38/JNK MAPK signaling pathways have been demonstrated to regulate a variety of cellular activities, including proliferation, differentiation, survival, and death [20, 26]. ERK/p38/JNK MAPK signaling can not only regulate cell cycle progression at different transition points but also modulate the cellular programmes for cell survival and differentiation[26, 27]. Once activated, ERK/p38/JNK MAPK could induce the levels of CDK6, CCND1 and CCNE1, and reduce the levels of p16, p21 and p27, thereby inducing G1/s and G2/M transition and cell proliferation [28-30]. Additionally, some studies reported that activation of ERK/p38/JNK MAPK resists apoptosis and promotes cell survival in HCC [27]. Here, we observed higher levels of p-ERK, p-p38 and p-JNK in HepG2 cells overexpressing DBH-AS1 as well as lower levels of p-EKR, p-p38 and p-JNK in Sh-DBH-AS1 Hep3B cells than control group separately. Our data suggests that ERK/p38/JNK MAPK signaling pathway can be activated by DBH-AS1 and may be partially responsible for DBH-AS1-induced cell proliferation and survival.

Given the results we obtained in clinical data analysis that the expression of DBH-AS1 is correlated to HBsAg, we wonder whether DBH-AS1 could be regulated by HBx protein. HBx has been demonstrated to promote liver cell proliferation and inhibit cell apoptosis via MAPK signaling [31, 32]. Therefore, we focused on the investigation of the relationship between HBx and DBH-AS1. Our findings confirmed that DBH-AS1 can be markedly induced by HBx protein in hepatocytes. Moreover, clinical data indicated that higher levels of DBH-AS1 were found in HCC patients with HBV infection than those without HBV infection.

Increasing evidence has shown that the transcript factor p53 plays an important role in cell cycle arrest, senescence and apoptosis, thus suppressing the development of various cancers [33]. Recent studies have characterized a series of p53-related lncRNAs, such as lincRNA-p21 [34], GAS5 [35], MEG3 [36], PVT1 [37], loc285194 [38]. Importantly, HBx protein has been reported to bind to and inhibit the expression of p53 and other tumour suppressor genes and senescence-related factors [39, 40]. Thus, we wonder whether there is a relationship between p53 and HBx-related lncRNA DBH-AS1 in HCC. Interestingly, we found a putative p53-binding site among the 2000-bp sequence upstream of DBH-AS1 gene and suppressed p53 expression resulted in elevated expression of DBH-AS1. But whether p53 can directly bind to the promoter region of DBH-AS1 still remains further investigated. We will next identify the promoter region of DBH-AS1 gene and use chromatin immunoprecipitation assays to further confirm the directly interaction between p53 and DBH-AS1 gene. Additionally, consistent with the finding reported by Matouk, I. J. [21] that a functional link exists between p53, hypoxia and lncRNA H19, we observed upregulation of DBH-AS1 under hypoxia stress, indicating that there might also be a link between p53 and DBH-AS1 under hypoxic stress.

Although we confirm that HBx-related lncRNA DBH-AS1 may act as an oncogene to promote cell proliferation and survival via MAPK signaling pathway, the molecules directly functioning downstream of DBH-AS1 still remain unclear. Consequently, further studies such as RNA-binding protein immunoprecipitation assays are necessary to find out the proteins or microRNAs directly binding with DBH-AS1. Additionally, it is of great necessity for us to collect adjacent HCC tissues or normal liver tissues and then compare the expression of DBH-AS1 between HCC tumor tissues and adjacent tissues or normal liver tissues.

In summary, our findings suggest that lncRNA DBH-AS1 promotes cell proliferation and survival via MAPK signaling in HCC. Modulation of the tumor proliferation effect through inhibiting MAPK activation mediated by DBH-AS1 overexpression might be used as a potential target for HCC prevention and therapy. Moreover, DBH-AS1 is found to be induced by HBx and inactivated by p53. We primarily confirm the regulatory mechanism of DBH-AS1 in HCC progression. Our findings demonstrate that DBH-AS1 is a potential oncogene participating in HCC pathogenesis.

## MATERIALS AND METHODS

### Cell lines and patient samples

The HepG2, SMMC-7721, Hep3B, MHCC97H, SK-Hep1 human HCC cell lines and the LO2, QSG7701 human immortalized normal hepatocytes were obtained from the Cell Bank of Type Culture Collection (Chinese Academy of Sciences, Shanghai, China). Cells were cultured in Dulbecco's modified Eagle's medium (DMEM, Gibco, Gaithersburg, MD, USA) containing 10% fetal bovine serum (FBS, Gibco) and incubated at 37°C in an atmosphere of 5% CO2. HCC tissues were obtained from patients who had undergone routine surgery from 2012 to 2014 at Nanfang Hospital, Southern Medical University. All tissues were histopathologically conﬁrmed as HCC. Written informed consent for the biological studies was obtained from each patient involved in the study, and the study was approved by the Ethics Committee of Nanfang Hospital.

### RNA extraction and real-time quantitative PCR analysis (qRT-PCR)

Total RNA was extracted from cultured cells or tissues using TRIzol Reagent (Takara, Dalian, China). For lncRNA DBH-AS1, first-strand cDNA was synthesized using the M-MLV Reverse Transcriptase (Promega, Madison WI, USA). For mRNAs, cDNA was generated using the PrimeScript RT reagent kit (Takara). The RNA expression levels were measured by qRT-PCR using SYBR Green PCR Master Mix (Takara) which was performed on the ABI 7500 Fast Real Time PCR system (Applied Biosystems, Foster City, CA, USA). U6 and β-actin were used as internal controls. All results were expressed as the means ± SD of at least three independent experiments. Comparative quantification was determined using the 2^−ΔΔCt^ method. The primers used are presented in Supplementary Table S1.

### Western blot analysis

Total proteins were prepared from the samples by complete cell lysis (Keygen Biotech, Jiangsu, China) with protease and phosphatase inhibitors. Identical quantities of proteins were separated on sodium dodecyl sulfate-polyacrylamide gel electrophoresis gels and transferred onto polyvinylidene difluoride membranes. After incubation with antibodies specific for HBx (Abcam, Cambridge, UK), CDK6 (Cell Signaling Technology, Beverly, MA, USA), CCND1(Cell Signaling Technology), CCNE1(Cell Signaling Technology), p16(Cell Signaling Technology), p21(Cell Signaling Technology), p27(Cell Signaling Technology), Caspase3(Immunoway, USA), cleaved Caspase3(Cell Signaling Technology), ERK (Cell Signaling Technology), p-ERK (Cell Signaling Technology), p38 (Cell Signaling Technology), p-p38 (Cell Signaling Technology), JNK(Cell Signaling Technology), p-JNK(Cell Signaling Technology), p53(Millipore, Schwalbach/Ts., Germany), HIF-1α(Abcam), GAPDH (Cell Signaling Technology) and β-actin (Proteintech, USA), the blots were incubated with goat anti-rabbit or anti-mouse secondary antibodies (Bioss, Beijing, China). The proteins were visualized using a chemiluminescence method (ECL Plus Western Blotting Detection System; Amersham Biosciences, Foster City, CA, USA) and quantified by ImageJ software.

### Construction of stable cell lines

To obtain cell lines stably overexpressing DBH-AS1, HepG2 and SMMC7721 cells were infected with the Lv-DBH-AS1 and Lv-control viruses (GeneChem, Shanghai, China). To observe the knockdown effects of DBH-AS1 *in vitro*, Hep3B and SK-Hep1 cells were transfected with the shRNA-DBH-AS1 (Sh-DBH-AS1) or control (Sh-con) viruses (GeneChem). Recombinant lentiviruses containing HBx (Lv-HBx) or control (Lv-con) purchased from LAND (Guangzhou, China) were used to infect HepG2 and LO2 cell lines. The infection efficiency was confirmed by qRT-PCR or western blot.

### Cell counting kit-8 (CCK-8) assay

The cell viability was assessed by CCK8 (Dojindo Laboratories, Kumamoto, Japan) according to the manufacturer's protocol. All of the experiments were performed in sixth. The cell proliferation curves were plotted using the absorbance at each time point.

### 5-ethynyl-2′-deoxyuridine (EdU) incorporation assay

EdU incorporation assay was carried out using the Cell-Light EdU imaging detecting kit (RiboBio, Guangzhou, China) according to the manufacturer's instructions. Briefly, cells were seeded in 96-well plates at a density of 5000 cells/well. After adherence, we added EdU labeling medium and incubated for about 60min. After fixed with 4% formaldehyde for 15 min and treated with 0.5 % Triton X-100 for 20 min, cells were exposed to Apollo reaction cocktail for 30 min. Subsequently, the DNA from cells in each well was stained with Hoechst 33342 (5 ug/mL) for 30 min and visualized under a fluorescent microscope.

### Colony formation assay

Cells were seeded in 6-well plates at a density of 100cells/well. After incubation for 14 days, cells were washed twice with PBS, fixed with methanol and stained with crystal violet. The number of colonies containing > 50cells was counted under a microscope.

### Cell cycle analysis

Cells were harvested and washed twice with PBS. After fixed in 70% ice-cold ethanol and kept overnight at 4°C, cells were stained with propidium iodide supplemented with RNaseA (Keygen Biotech) for 30 min at 37°C. The DNA content of labeled cells was acquired using FACS cytometry (BD Biosciences Inc., Franklin Lakes, NJ, USA). Each experiment was performed in triplicate.

### Cell apoptosis analysis

Cells were cultured in serum-free medium for 48h. Then, the apoptosis assay was done with the Annexin V-7AAD apoptosis detection kit (Keygen Biotech) according to the manufacturer's instructions. Cells were then analyzed by FACS cytometry (BD Biosciences Inc.).

### *In vivo* tumor growth assay

All investigations were approved by the Animal Experimental Committee of Nanfang Hospital. The male BALB/C nude mice were purchased from the Guangdong Experimental Animal Center of the Chinese Academy of Sciences, bred and maintained in a specific pathogen-free facility. For xenograft models: cells were collected by trypsinization, washed twice with PBS, and resuspended with serum-free medium. A total of 5 × 10^6^ SMMC-7721 cells stably transfected with Lv-DBH-AS1 or Lv-control in 0.1 ml DMEM medium was independently injected subcutaneously into the left back and right back of 9 nude mice. Tumor size was monitored routinely for 5 weeks. Mean percent of body weight (±SEM) and tumor size for each group was measured every 7 days.

### Immunohistochemistry

Immunohistochemistry for Ki-67 was performed on paraffin sections using a primary antibody against Ki67 (Abcam) and a horseradish peroxidase-conjugated rabbit anti-goat antibody (Maixin, Fuzhou, China), and the proteins in situ were visualized with 3, 3-diaminobenzidine and analyzed using a bright field microscope.

### RNA interference and transfection

HepG2 and LO2 cells were plated in a 6-well plate at a 30-50% confluence. After 24h, siRNA against TP53 (Ambion, Carlsbad, CA) were transfected into HepG2 and LO2 cells using Lipofectamine 2000 reagent (Invitrogen, USA) according to the manufacturer's instructions. Cells transfected with the transfection agent, but no siRNA (Mock) or scramble-control siRNA (NC) were used as controls. The cells were harvested 48 hours after transfection.

### Statistical analysis

SPSS 13.0 software (SPSS Inc., Chicago, IL, USA) and GraphPad software (GraphPad Software, Inc., La Jolla, CA, USA) were used to analyze all data for statistical significance. The Chi-Square test was applied to the examination of relationship between lncRNA DBH-AS1 expression levels and clinicopathological characteristics. Two-tailed Student's *t*-test was used for comparisons of two independent groups. One-way ANOVA was used to determine the differences between groups for all in vitro analyses. Statistical signiﬁcance was set at *P < 0.05, **P < 0.01, ***P < 0.001. *P* < 0.05 was considered statistically signiﬁcant.

## SUPPLEMENTARY MATERIAL FIGURE AND TABLE



## Abbreviations

HCC: hepatocellular carcinoma
HBV: hepatitis B virus
HBx: hepatitis B virus x protein
lncRNA: long non-coding RNA
HBsAg: hepatitis B surface antigen
qRT-PCR: quantitative real-time polymerase chain reaction
FACS: fluorescence-activated cell sorting
siRNA: small interfering RNA
MAPK: mitogen-activated protein kinase
ERK: extracellular signal-regulated kinase;
JNK: c-Jun N-terminal kinase.
