# Supplementary material for: HBx-related long non-coding RNA DBH-AS1 promotes cell proliferation and survival by activating MAPK signaling in hepatocellular carcinoma
Source: Oncotarget. 2015 Sep 15;6(32):33791–804. doi: 10.18632/oncotarget.5667 (PMC4741803; doi:10.18632/oncotarget.5667)
Supplement: Supplementary file 1 [file oncotarget-06-33791-s001.pdf]

HBx-related long non-coding RNA DBH-AS1 promotes cell proliferation and survival by activating MAPK signaling in hepatocellular carcinoma

Supplementary Material

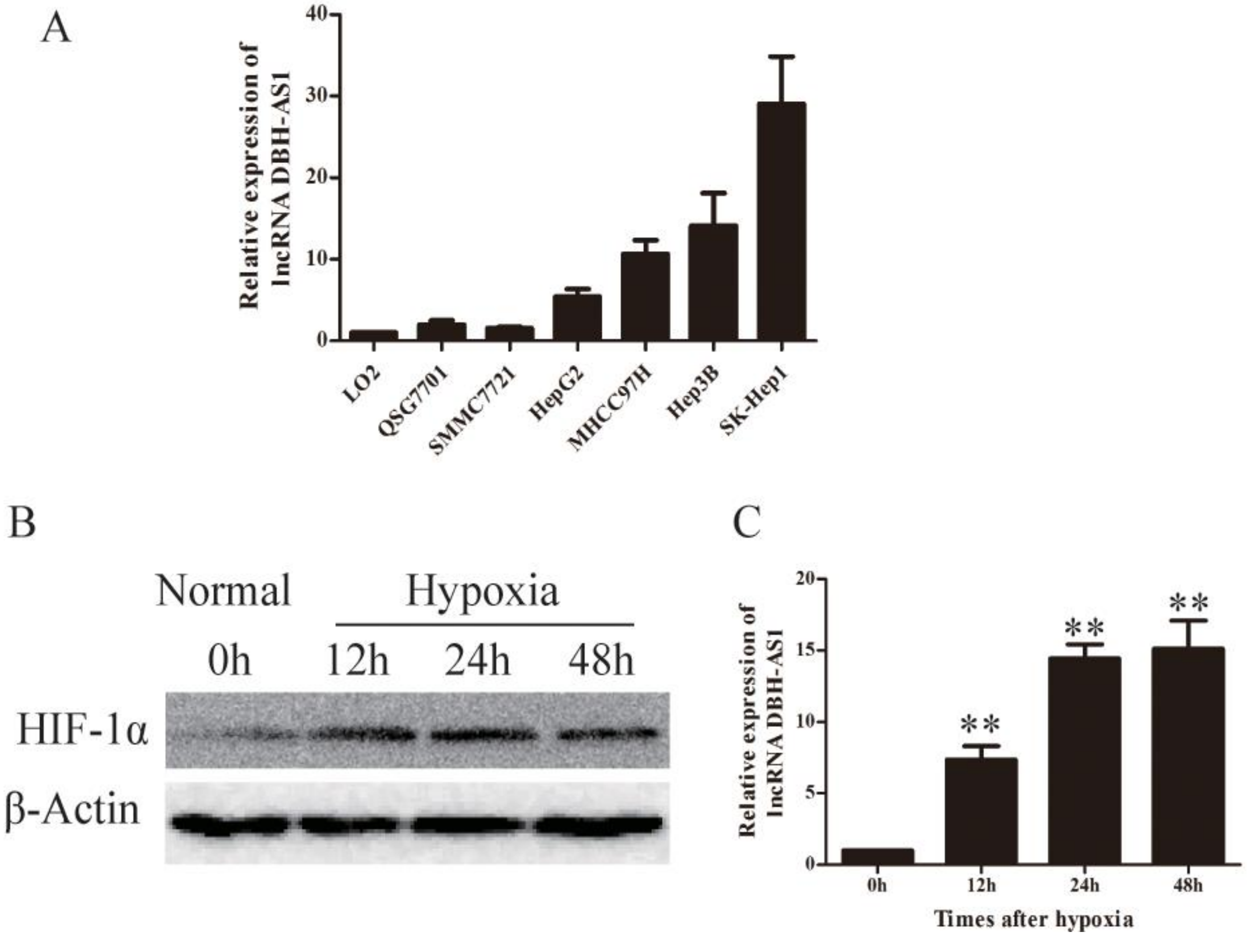

Figure S1 (A) The expression of DBH-AS1 was measured in several liver cancer cell lines and hepatic immortal cell lines. The cell lines were compared with LO2 cells, a hepatic immortal cell line. **(B)** Western blot analysis of HIF-1 α protein expression after 12h, 24h, 48h of hypoxia. **(C)** qRT-PCR analysis of DBH-AS1 expression after 12h, 24h and 48h of hypoxia. Mean values are from at least three independent experiments. **P*<0.05, ***P*<0.01, ****P*<0.001.

Table S1: Sequences of primers and shRNA used in this study.

Name		Sequences
**qRT-PCR primers**		
**lncRNA DBH-AS1**	sense	CGTCCACTCGTCTGTTCACT
	antisense	TAACACCCCATCCGCTTGT
**P16**	sense	GGGTTTTCGTGGTTCACATCC
	antisense	CTAGACGCTGGCTCCTCAGTA
**P21**	sense	TGTCCGTCAGAACCCATGC
	antisense	AAAGTCGAAGTTCCATCGCTC
**P27**	sense	AACGTGCGAGTGTCTAACGG
	antisense	CCCTCTAGGGGTTTGTGATTCT
**CDK6**	sense	TCTTCATTCACACCGAGTAGTGC
	antisense	TGAGGTTAGAGCCATCTGGAAA
**CCND1**	sense	GCTGCGAAGTGGAAACCATC
	antisense	CCTCCTTCTGCACACATTTGAA
**CCNE1**	sense	AAGGAGCGGGACACCATGA
	antisense	ACGGTCACGTTTGCCTTCC
**HBx**	sense	CGTGTGCACTTCGCTTCA
	antisense	CCAACTCCTCCCAGTCTTTAA
**TP53**	sense	CAGCACATGACGGAGGTTGT
	antisense	TCATCCAAATACTCCACACGC
**β-actin**	sense	TGGCACCCAGCACAATGAA
	antisense	CTAAGTCATAGTCCGCCTAGAAGCA
**U6**	sense	CTCGCTTCGGCAGCACA
	antisense	AACGCTTCACGAATTTGCGT
**shRNA sequences**		
**sh-DBH-AS1**	CCGGCAGGAAATTCGTATCAGGTACCTCGAGGTACCTGATACGAATTTCCTGTTTTTG	
